# Independent replication of advanced brain age in mild cognitive impairment and dementia: detection of future cognitive dysfunction

**DOI:** 10.1038/s41380-022-01728-y

**Published:** 2022-08-16

**Authors:** Helmet T. Karim, Howard J. Aizenstein, Akiko Mizuno, Maria Ly, Carmen Andreescu, Minjie Wu, Chang Hyung Hong, Hyun Woong Roh, Bumhee Park, Heirim Lee, Na-Rae Kim, Jin Wook Choi, Sang Won Seo, Seong Hye Choi, Eun-Joo Kim, Byeong C. Kim, Jae Youn Cheong, Eunyoung Lee, Dong-gi Lee, Yong Hyuk Cho, So Young Moon, Sang Joon Son

**Affiliations:** 1 Department of Psychiatry, University of Pittsburgh School of Medicine, Pittsburgh, PA USA; 2 Department of Bioengineering, University of Pittsburgh, Pittsburgh, PA USA; 3 Department of Psychiatry, Ajou University School of Medicine, Suwon, Republic of Korea; 4 Department of Biomedical Informatics, Ajou University School of Medicine, Suwon, Republic of Korea; 5 Office of Biostatistics, Medical Research Collaborating Centre, Ajou Research Institute for Innovative Medicine, Ajou University Medical Centre, Suwon, Republic of Korea; 6 Department of Radiology, Ajou University School of Medicine, Suwon, Republic of Korea; 7 Department of Neurology, Samsung Medical Centre, Sungkyunkwan University School of Medicine, Seoul, Republic of Korea; 8 Department of Neurology, Inha University College of Medicine, Incheon, Republic of Korea; 9 Department of Neurology, Pusan National University Hospital, Pusan National University School of Medicine and Biomedical research institute, Busan, Republic of Korea; 10 Department of Neurology, Chonnam National University Medical School, Gwangju, Republic of Korea; 11 Department of Gastroenterology, Ajou University School of Medicine, Suwon, Republic of Korea; 12 Human Genome Research and Bio-Resource Centre, Ajou University Medical Centre, Suwon, Republic of Korea; 13 Department of Neurology, Ajou University School of Medicine, Suwon, Republic of Korea

**Keywords:** Predictive markers, Diagnostic markers, Prognostic markers

## Abstract

We previously developed a novel machine-learning-based brain age model that was sensitive to amyloid. We aimed to independently validate it and to demonstrate its utility using independent clinical data. We recruited 650 participants from South Korean memory clinics to undergo magnetic resonance imaging and clinical assessments. We employed a pretrained brain age model that used data from an independent set of largely Caucasian individuals (*n* = 757) who had no or relatively low levels of amyloid as confirmed by positron emission tomography (PET). We investigated the association between brain age residual and cognitive decline. We found that our pretrained brain age model was able to reliably estimate brain age (mean absolute error = 5.68 years, *r*(650) = 0.47, age range = 49–89 year) in the sample with 71 participants with subjective cognitive decline (SCD), 375 with mild cognitive impairment (MCI), and 204 with dementia. Greater brain age was associated with greater amyloid and worse cognitive function [Odds Ratio, (95% Confidence Interval {CI}): 1.28 (1.06–1.55), *p* = 0.030 for amyloid PET positivity; 2.52 (1.76–3.61), *p* < 0.001 for dementia]. Baseline brain age residual was predictive of future cognitive worsening even after adjusting for apolipoprotein E e4 and amyloid status [Hazard Ratio, (95% CI): 1.94 (1.33–2.81), *p* = 0.001 for total 336 follow-up sample; 2.31 (1.44–3.71), *p* = 0.001 for 284 subsample with baseline Clinical Dementia Rating ≤ 0.5; 2.40 (1.43–4.03), *p* = 0.001 for 240 subsample with baseline SCD or MCI]. In independent data set, these results replicate our previous findings using this model, which was able to delineate significant differences in brain age according to the diagnostic stages of dementia as well as amyloid deposition status. Brain age models may offer benefits in discriminating and tracking cognitive impairment in older adults.

## Introduction

Models of the average ageing process are becoming prevalent in multiple fields, and for a decade, brain ageing markers have been used to identify important neuroanatomical differences in various disorders. These markers may provide individualised risk-assessments and predictions for age-associated neurodegenerative diseases [1]. There is a shift towards identifying individual, rather than average, differences that may provide tailored predictions for long-term health outcomes [2]. Brain age is based on machine learning to estimate an individual’s chronological age from neuroimaging data [1–7]. Individuals whose brain structures are estimated to be older than age-matched healthy peers may have experienced a higher cumulative exposure to factors that are associated with brain atrophy, were more impacted by those pathologic factors, or alternatively reflect non-neurodegenerative processes [8].

Recently, these models have been used to demonstrate the association between greater brain age and cognitive impairment, Alzheimer’s disease (AD), traumatic brain injury, and mortality [3, 4, 6, 9, 10]. Given the relationship between ageing and disease, there could be common underlying mechanisms. Concerning complex brain diseases, combining ageing-related biomarkers with more disease-specific biomarkers can lead to further improvements in diagnostic and prognostic modelling of neurodegenerative disease [4]. Thus, brain age prediction approaches may improve the assessment of individual risk for neurodegenerative diseases, guide diagnostics and personalised interventions [1, 2, 11]. Ideally, individuals identified as having low cognitive function using traditional cognitive batteries, undergo brain magnetic resonance imaging (MRI) to estimate their brain age which can then be used to stratify risk for future cognitive impairment; this can also be determine if a more rigorous schedule of assessments needs to be done and identify possible treatments to alter brain age and its trajectories. Additionally, those at heighted risk may be considered for amyloid positron emission tomography (PET) scans, especially in younger individuals. Given that brain age models utilise grey matter, they make up the ‘neurodegeneration’ component of the Amyloid-Tau-Neurodegeneration (ATN) model thus it is a natural progression to develop models that reflect this component.

We have previously trained a machine learning model for estimating brain age using grey matter volume in a sample of healthy individuals without significant brain amyloid [3]. We showed that this model predicted brain age that was greater in amyloid-positive compared to amyloid-negative individuals [3]. We additionally showed that individuals with worse cognitive function (e.g. AD) had greater brain age compared to those with mild cognitive impairment (MCI) or non-demented controls [3]. We have shown preliminary feasibility in identifying advanced brain age and its association with worry [11], rumination [11], and chronic back pain [12]; however, the initial training model has not yet been independently validated in a different ethnic population and clinical setting. Moreover, the longitudinal changes in cognitive function using this brain age model have not yet been evaluated.

In this study, we aimed to independently validate the clinical utility of this previously trained model for predicting future cognitive decline in a new cohort. As another novel aspect of the present study, we applied the brain age model, which was trained primarily with Caucasian samples, to non-Caucasian data. We analysed data from a large sample of South Korean participants with subjective cognitive decline (SCD), MCI, and dementia including AD who had amyloid PET scans, apolipoprotein E (APOE) measurements, cognitive testing, and clinical data. We tested the following confirmatory hypotheses: (1) participants with cognitive impairment will have higher brain age than cognitively normal older adults; (2) greater brain age will be associated with worse cognitive function and disability at study entry; (3) individuals who are amyloid positive will have greater brain age compared to those who are amyloid negative. We will show clinical utility by testing the following hypothesis: (4) baseline brain age predicts future cognitive decline better than baseline chronological age, APOE status, baseline amyloid levels, baseline medial temporal lobe volume, and even baseline cognitive function.

## Methods

### Participants

This study was a part of the ongoing Biobank Innovations for chronic Cerebrovascular disease With ALZheimer’s disease Study (BICWALZS) and the Centre for Convergence Research of Neurological Disorders. The BICWALZS was planned and initiated in October 2016 by the Korea Disease Control and Prevention Agency for the Korea Biobank Project, which is a national innovative biobanking program to foster biomedical and healthcare research and development infrastructure. Memory clinics of five university hospitals and a community geriatric mental health centre were involved in this study. Participants were recruited voluntarily from those who visited these neurology or psychiatry memory out-patient clinics. The original goal was to facilitate, regulate, and ensure optimal use of human biological specimens for research from real-world data in the fields of SCD, MCI, AD and subcortical vascular dementia (SVaD).

The clinical diagnosis criteria used for this study were as follows: SCD criteria included self-and/or informant reports of cognitive decline, but no objective impairment in cognitive tasks [no less than −1.5 SD in each of neurocognitive test domain and Clinical Dementia Rating (CDR) = 0] [13]; patients with MCI were evaluated based on a CDR [14] score of 0.5 and the expanded Mayo Clinic criteria [15]; patients with AD dementia were evaluated using the National Institute on Aging-Alzheimer’s Association core clinical probable AD dementia criteria [16]; and SVaD was evaluated based on above-moderate white matter hyperintensity (WMH) and vascular dementia criteria in accordance with the Diagnostic Statistical Manual of Mental Disorders, fifth edition [17]. Patients with a history of neurological or medical conditions, such as territorial cerebral infarction, intracranial haemorrhage, Parkinson’s disease, heart failure, renal failure, or others that could interfere with the study were excluded.

The BICWALZS is registered with the Korean National Clinical Trial Registry (Clinical Research Information Service; identifier, KCT0003391). The study was approved by the Institutional Review Board of Ajou University Hospital (AJIRB-BMR-SUR-16-362). Written informed consent was obtained from all participants and caregivers. Participants from the BICWALZS were recruited at the memory clinics of seven university-affiliated hospitals and community geriatric centres in South Korea. All participants were Korean (Eastern Asian ethnicity). None of the participants in this study was a part of the initial training sample of our previously trained model [3]. We identified 687 participants (age range = 49–89 year; 80 SCD, 389 MCI and 218 dementia) with 3D T1-weighted brain MRI from 2016 to 2020 and estimated each individual’s brain age. Among these individuals, we used data from 650 participants (age range = 49–89 years; 71 SCD, 375 MCI and 204 dementia) with available amyloid PET information for clinical validation. Three hundred and thirty-six participants were followed up for cognitive decline by annual assessment of clinical diagnosis and Clinical Dementia Rating Sum of Box (CDR-SB) [14]. Among them, 284 subjects had less than the score of CDR 0.5 and 240 subjects were diagnosed with SCD or MCI at baseline, indicating cognitively none or only mild impairment [18]. Duration of follow-up was 19.69 ± 8.66, 19.38 ± 8.44 and 19.44 ± 8.47 months, respectively.

### Clinical and biological assessment

Clinical and biological assessments are described in the supplement. Briefly, we collected data on neurocognitive battery including standardised tests for language, visuospatial abilities, memory, and frontal/executive function [19]. The participants underwent ^18^F-flutemetamol PET scanning. To quantify ^18^F-flutemetamol retention, the standard uptake value ratio (SUVR) was obtained using the pons as a reference region. Informed consent was obtained from all participants regarding the collection and genotyping of blood genomic DNA and APOE genotyping was obtained. MRI T1 coronal images were used for the visual assessment and both left and right medial temporal lobe atrophy (MTA) were visually rated separately.

### Brain age estimation

Methods for MRI acquisition and structural processing are described in the supplement. Sequence parameters were reported in Supplementary Table 1. We have previously validated a brain age estimation algorithm that predicts chronological age with grey matter volume [3] using the Pattern Recognition for Neuroimaging Toolbox [20]. Whole brain, voxel-wise grey matter volume maps were mean-centered and used to calculate a similarity matrix kernel [21] that was input into a Gaussian processes regression to predict chronological age. The training set, which included 757 adult MRIs of individuals without any psychiatric or neurologic disorder as well as Alzheimer’s pathology as measured by PET, has been previously described [3]. These data were from the Alzheimer’s Disease Neuroimaging Initiative, Information eXtraction from Images, and Open Access Series of Imaging Studies (OASIS-3) which are all publicly available. The cohort source was used as a covariate to account for differences in scanner, site and protocol. Site effect was not added as a feature when brain age was calculated as these data were not used as part of training, thus would not affect the model itself. Site effect was added as covariate to subsequent statistical models when modelling the association between brain age and clinical variables. The participants of this present study were not part of the training set. Using this pretrained model, we estimated the brain age of each participant in the present study (estimated brain age of the 687 participants is detailed in Supplementary Table 2. We used the data of 650 participants with amyloid PET information for clinical validation). While WMH might likely be a factor that influenced brain ageing, our brain age model utilises primarily grey matter and not white matter data. Thus, our brain age marker could more accurately be described as ‘grey matter’ age [11]. We additionally adjusted for the intercept and slope (i.e., subtract the intercept and divide by the slope) of the original brain age model, as this has been found to bias the brain age predictions.

### Statistical analysis

We conducted analyses using IBM SPSS 25 (IBM Corp., Armonk, NY, USA) and R software (version 4.1.1; R Foundation for Statistical Computing, Vienna, Austria). Scatter and violin plots were plotted using the R package ggplot2. Survival curves were plotted using the R package Survminer.

We checked the violation of the assumption of homoscedasticity by investigating skewness and kurtosis. We used the brain age residual as an index of age-related brain health. Brain age residual is the residual error after regressing out age, age squared, and sex onto brain age [brain age = intercept + β1(age centred, individual chronological age-mean of chronological age) + β2(age centred^2^) + β3(sex)+brain age residual]. Thus, high brain age residual represents greater brain age than expected at that chronological age adjusting for sex. This residual was calculated for every 650 participants. To examine whether the brain age residual was associated with clinical factors, analysis of covariance, linear and multinomial logistic regression analyses were conducted with the following dependent variables: CDR-SB, neurocognitive tests scores, and diagnosis of dementia while adjusting for age centred, age centred squared, sex, education, intracranial volume, study site, amyloid PET positivity and APOE e4 allele status. Using linear regression, we then investigated the association between brain age (dependent variable) and the following independent variables: age, age squared, sex, education, APOE e4, amyloid PET positive, WMH severity and lacunae.

To validate the value of the brain age residual in identifying participants with and without a diagnosis of dementia, the receiver operating curve characteristic (ROC) curve and sensitivity and specificity levels were evaluated in comparison with the traditional measures such as Mini Mental Status Examination (MMSE), amyloid PET SUVR adjusting for age centred, age centred squared, sex, education, intracranial volume, study site and APOE e4 allele status. The cut-off score was determined based on maximal sensitivity and specificity.

We then conducted survival analysis, using a Cox proportional hazards regression and Kaplan–Meier estimator in individuals who had available follow-up cognitive data (*N* = 366) to examine the capacity of brain age as a predictive marker of the progression of cognitive decline. We also conducted similar analyses in subgroups, primarily among participants who were normal or only mildly impaired at baseline (subgroup I: less than 0.5 point of CDR at baseline assessment, *N* = 284; subgroup II: diagnosed with SCD or MCI at baseline assessment, *N* = 240), and at this stage, it was important to note whether they progressed to the level of dementia [22]. This analysis tested whether the brain age residual at baseline predicted time-to-progression of cognitive decline. These analyses included several covariates including age centred, age-centred squared, sex, education, intracranial volume, study site, baseline CDR-SB, APOE e4 allele and amyloid PET positive. In these analyses, we defined two prime events: 1) the last follow-up when the CDR-SB score went beyond the known confidence intervals (CI) of the annual rate of change [annual rate of change (slope, 95% CI) in CDR-SB was known as 1.88(1.77–2.05) in those who progressed to CDR 1 from baseline CDR 0 or 0.5] [23] for total and subgroup I samples and 2) incident of clinical diagnosis with dementia for subgroup II sample. We also calculated absolute standardised hazard ratios [24] to compare brain age residual with MTA [25] and MMSE [26] as predictors for future cognitive decline. In addition, we used likelihood tests to compare nested Cox models. The z-transformation was applied to normalise the continuous variables such as MMSE and brain age residual in these analyses.

## Results

### Demographic characteristics and brain age prediction performance

**Fig. 1:**
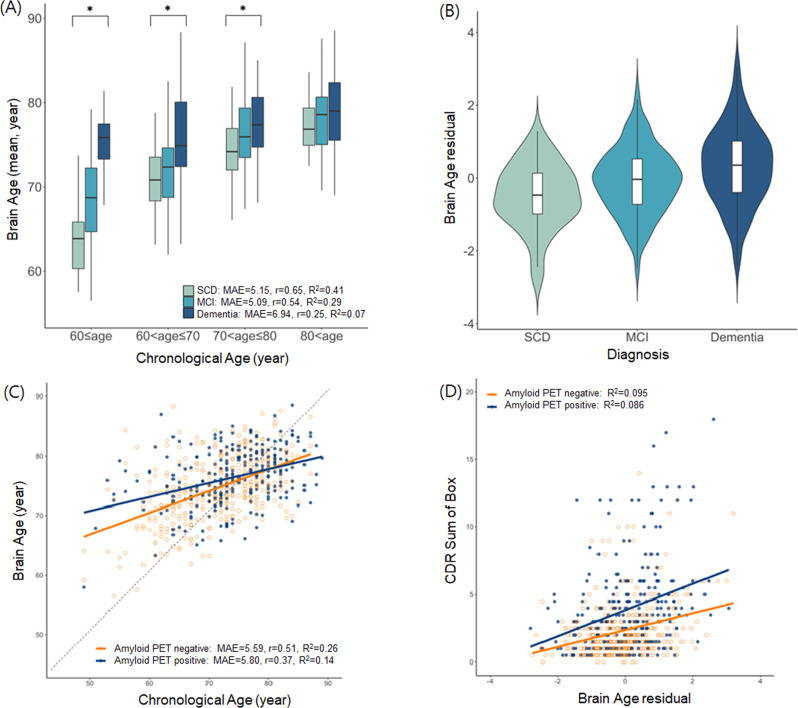
Characteristics of brain age according to clinical diagnosis and amyloid deposition status at baseline in all participants. **A** Association between brain and chronological ages according to clinical diagnosis. **B** Distribution of brain age residual according to clinical diagnosis. **C** Association between brain and chronological ages according to the presence of amyloid deposition. **D** Association between brain age residual and the CDR-SB score according to the presence of amyloid deposition. Brain age residual was calculated as (brain age = intercept + β1[age centred] + β2[age centred squared] + β3[sex] + brain age residual). * Analysis of variance were conducted (*p* < 0.05). Abbreviations: SCD subjective cognitive decline, MCI mild cognitive impairment, MAE mean absolute error, CDR-SB Clinical Dementia Rating Sum of Box, PET positron emission tomography.

We report the characteristics of the sample in Table 1, Fig. 1 and Supplementary Table 3. The mean age of baseline participants was 72.49 ± 7.54 years, and their brain age was 75.34 ± 5.16 years. The proportion of participants with a clinical diagnosis of MCI and dementia was 57.7% and 31.4%, respectively, and 77.8% showed cognitive impairment below the global CDR of 0.5.

**Table 1:** Clinical characteristics of study participants.

	Total baseline sample (*N* = 650)	
	Mean or *n*	SD or %
Brain age, mean (SD), years	75.34	5.16
Age, mean (SD), years	72.49	7.54
Education, mean (SD), years	8.11	4.85
Female, *n* (%)	438	67.40
Comorbidity, *n* (%)		
Hypertension	351	54.00
Diabetes mellitus	145	22.30
Hyperlipidemia	248	38.20
Cardiovascular disease	39	6.00
CDR, *n* (%)		
0	13	2.00
0.5	493	75.80
1	114	17.50
2 or more	30	4.60
CDR-Sum of Box score, mean (SD)	2.96	2.72
Clinical diagnosis, *n* (%)		
SCD	71	10.90
MCI	375	57.70
AD	137	21.10
SVaD	40	6.20
Other dementia	27	4.10
APOE genotype, *n* (%)		
E2/E2	1	0.20
E3/E2	74	11.40
E3/E3	380	58.50
E4/E2	13	2.00
E4/E3	160	24.60
E4/E4	22	3.40
Amyloid PET positive, *n* (%)	255	39.20
Global amyloid SUVR score, mean (SD)	0.69	0.17

*SD* standard deviation, *APOE* apolipoprotein E, *CDR* clinical dementia rating, *SCD* subjective cognitive decline, *MCI* mild cognitive impairment, *AD* Alzheimer’s disease, *SVaD* subcortical vascular dementia, *PET* positron emission tomography, *SUVR* standardised uptake value ratio.

The brain age prediction model was accurate with mean absolute error (MAE) = 5.68 years, *r*(650) = 0.47; *R*^2^ = 0.22 in the total 650 sample. In previous Caucasian test set, the model accuracy was MAE = 4.65 years, *r*(490) = 0.60; *R*^2^ = 0.36 [3]. Unlike our previous data set, our data contained many individuals with dementia who were expected to have greater than average MAE. The model was more accurate in the participants with SCD or MCI with MAE = 5.10 years, *r*(446) = 0.57; *R*^2^ = 0.32. Regarding amyloid negative normal or SCD subject, the model performances were MAE = 3.70 years, *r*(50) = 0.64; *R*^2^ = 0.36 in the previous Caucasian test set [3] and MAE = 5.09 years, *r*(63) = 0.69; *R*^2^ = 0.46 in the BICWALZS (Table 2). Considering the performance of previous study [4, 27, 28], our model was able to predict chronological age accurately within expected tolerance. The correlation map of brain age residual with grey matter volume using voxel-wise analysis was shown as the features for predicting brain age in Supplementary Fig. 1.

**Table 2:** Performance metrics calculated in a BICWALZS test set (*n* = 650)^a^.

	Total (*n* = 650)	Amyloid negative & SCD (*n* = 63)	Amyloid negative, SCD & MCI (*n* = 327)	Diagnosis				Amyloid deposition	
				SCD (*n* = 71)	MCI (*n* = 375)	SCD& MCI (*n* = 446)	Dementia (*n* = 204)	Negative (*n* = 395)	Positive (*n* = 255)
Age year, mean ± SD (range)	72.49 ± 7.54 (49-89)	69.51 ± 7.93 (49-84)	71.07 ± 7.24 (49-85)	69.80 ± 7.72 (49-84)	72.49 ± 7.06 (49-89)	72.07 ± 7.23 (49-84)	73.42 ± 8.13 (51-88)	71.51 ± 7.39 (49-87)	74.00 ± 7.54 (49-89)
MAE, mean ± SD	5.68 ± 4.74	5.09 ± 3.60	5.32 ± 4.03	5.15 ± 3.55	5.09 ± 4.07	5.10 ± 3.99	6.94 ± 5.86	5.59 ± 4.59	5.80 ± 4.96
RMSE, mean ± SD	7.40 ± 9.66	6.24 ± 6.89	6.68 ± 7.96	6.25 ± 6.82	6.52 ± 8.23	6.48 ± 8.05	9.09 ± 11.49	7.24 ± 9.47	7.63 ± 9.92
*r*	0.47^*^	0.69^*^	0.58^*^	0.65^*^	0.54^*^	0.57^*^	0.25^*^	0.51^*^	0.37^*^
*R*^2^	0.22	0.46	0.33	0.41	0.29	0.32	0.07	0.26	0.14

*SD* standard deviation, *SCD* subjective cognitive decline, *MCI* mild cognitive impairment, *MAE* mean absolute error, *RMSE* root mean square error, *MRI* magnetic resonance imaging, *PET* positron emission tomography.

*p < 0.001.

^a^Estimated brain age of 650 participant with both available 3DT1 weighted MRI and amyloid PET data.

The brain age of the non-dementia group showed a relatively smaller deviation from its chronological age compared with the dementia group, as illustrated in Fig. 1A and Supplementary Fig. 2, with the line of best fit closely aligned to the reference line (i.e., brain age = chronological age) but less so for the dementia group. In addition, the gap between brain and chronological age in dementia group was more prominent at the early age bins.

### Associations between brain age and clinical diagnosis and measures of clinical symptoms

Greater cognitive impairment severity, measured by CDR-SB, was associated with a greater brain age residual. Even after correcting for multiple comparisons (using false discovery rate correction), multiple measures were significantly associated with the brain age residual, including the association between worse cognitive function with greater brain age residual (*B* = 0.77, *p* < 0.001). Among the association between neurocognitive test items and brain age residual, Boston naming (*B* = −0.35, *p* < 0.001), Complex Figure copy (*B* = −0.57, *p* < 0.001) and Stroop test (*B* = −0.52, *p* < 0.001) were relatively high. Details of the linear regression results for associations of brain age residual and cognitive function are described in Supplementary Table 4. Participants with dementia showed a greater brain age residual (0.33 ± 1.08) compared with those with SCD (−0.50 ± 0.89) and MCI (−0.08 ± 0.92), even after adjusting for age centred, age centred square, education, intracranial volume, and study site (analysis of covariance: *F* = 21.09, *R*^2^ = 0.07, *p* < 0.001) (Fig. 1B). In the regression model with brain age as the dependent variable, we found that greater amyloid deposition was significantly associated with greater brain age as was chronological age and male sex compared to female sex (Fig. 1C, Supplementary Table 5, and Supplementary Fig. 3).

### Multinominal logistic regression and ROC curve analysis for current cognitive impairment

Using multinomial logistic regression, we found that greater brain age was associated with a higher odds ratio (OR) for MCI and dementia compared to that for individuals with SCD [OR (95% CI) for MCI = 1.54 (1.13–2.12); OR (95% CI) for dementia = 2.52 (1.76–3.61)] (Table 3). The OR of the brain age residual for MCI and dementia was not affected by demographic factors, APOE e4 status, or amyloid deposition status. Using a similar approach, we found that greater brain age was associated with a higher OR of amyloid positivity compared to amyloid negativity even after adjusting for similar factors [OR (95% CI) for amyloid positivity = 1.28 (1.06–1.55)] (Table 3).

**Table 3:** Association of the brain age residual with MCI, dementia and amyloid deposition assessed by multinominal logistic regression.

Reference: SCD (*n*/*N* = 71/650)	MCI (*n*/*N* = 375/650)				Dementia (*n*/*N* = 204/650)			
	OR	95% CI		*p* value (FDR correction)	OR	95% CI		*p* value (FDR correction)
Model 1	1.57	1.20	2.06	0.004	2.43	1.80	3.27	<0.001
Model 2	1.51	1.12	2.03	0.017	2.37	1.71	3.29	<0.001
Model 3	1.54	1.13	2.09	0.018	2.57	1.82	3.63	<0.001
Model 4	1.54	1.13	2.12	0.018	2.52	1.76	3.61	<0.001

Reference: Amyloid PET negative(*n*/*N* = 395/650)	Amyloid PET positive (*n*/*N* = 255/650)							
	OR			95% CI				*p* value (FDR correction)
Model 1	1.19			1.02	1.40			0.030
Model 2	1.22			1.03	1.46			0.030
Model 3	1.28			1.06	1.55			0.030

*SCD* subjective cognitive decline, *MCI* mild cognitive impairment, *OR* odds ratio, *CI* confidence interval, *APOE* apolipoprotein E, *PET* positron emission tomography.

Brain age residual was calculated as (brain age = intercept + β1[age centred] + β2[age centred squared] + β3[sex] + brain age residual), and multinomial logistic regression was conducted.

Model 1: Brain age residual (continuous variable); Model 2: Model 1 + age centred, age centred squared, sex, education, intracranial volume, and study site; Model 3: Model 2 + APOE e4; Model 4: Model 3 + amyloid PET positive.

To demonstrate the association between cognitive status and brain age residual, we conducted an ROC analysis in three groups (total sample, under 77, and under 70 years) based on tertile values. In the total baseline sample, the ROC analysis of the brain age residual for dementia resulted in an area under the curve (AUC) of 0.761, sensitivity of 0.686 and specificity of 0.716. The AUC value of brain age was lower than that of MMSE (AUC of 0.876) or amyloid PET (AUC of 0.785), but similar to the value of the cross-validation test set in the previous study (AUC of 0.710) [3]. In the subgroup analyses by age, the brain age residual resulted in an AUC of 0.782 (under 77 years) and 0.870 (under 70 years) respectively, showing a trend for better performance for a classification in younger individuals (all models are described in Supplementary Table 6 and Supplementary Fig. 4).

### Initial brain age residual predicts longitudinal cognitive worsening

**Fig. 2:**
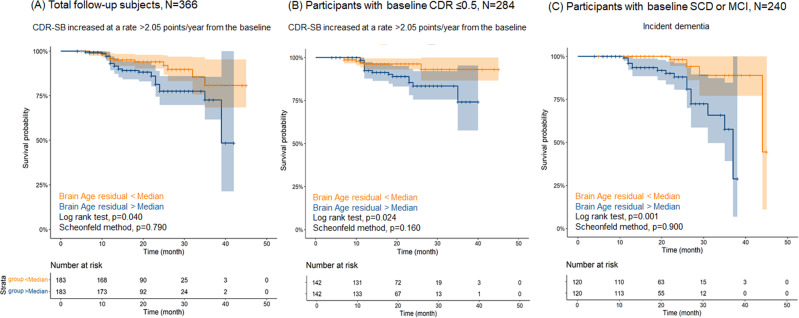
Kaplan–Meier plot for time-to-event comparison between individuals with higher and lower brain age residuals among follow-up participants^ǂ^. Brain age residual was calculated as (brain age = intercept + β1[age centred] + β2[age centred squared] + β3[sex] + brain age residual) in these samples. *Cognitive end point (time-to-event) was defined when the CDR-SB score increased at a rate >2.05 points/year from the baseline CDR-SB score at the final follow-up (Annual rate of change (slope, 95% CI) in CDR-SB was known as 1.88(1.77-2.05) in those who progressed to CDR 1 from baseline CDR 0 or 0.5) [23]. ǂLog rank tests were conducted for the follow-up participants. Abbreviations: CDR Clinical Dementia Rating, CDR-SB Clinical Dementia Rating Sum of Box.

Of the entire sample, 366 participants were followed up for cognitive function through annual CDR-SB assessment. Among them, 284 participants had a CDR less than 0.5 and 240 participants were diagnosed with SCD or MCI at baseline assessment. Their mean follow-up durations were 19.69 ± 8.66, 19.38 ± 8.44 and 19.44 ± 8.47 months, respectively. Detailed characteristics of these samples are described in Supplementary Tables 7 and 8. We investigated two cognitive endpoints: 1) increased CDR-SB score at a rate >2.05 points/year from the baseline CDR-SB score at the final follow-up for the total sample and 284 subsets and 2) dementia incidence for the 240 subsets. Using a Cox proportional-hazards regression model, the hazard ratios (HRs) in the total sample (366 participants) of the brain age residual for cognitive endpoint was 1.94 (1.33–2.81, *p* = 0.001). In the 284 and 240 subsets, the HRs were 2.31 (1.44–3.71, *p* = 0.001) and 2.40 (1.43–4.03, *p* = 0.001), respectively. These results were statistically significant even after adjusting for APOE e4 and amyloid PET positivity. Detailed results of Cox proportional hazards regression and the Kaplan–Meier estimator are shown in Table 4 and Fig. 2. Using an absolute standardised HR, we investigated MTA, MMSE and amyloid PET positivity as predictors of cognitive decline or incident dementia and found that brain age residual [HR (95% CI) = 2.31 (1.44–3.71), *p* = 0.001] was able to predict future cognitive decline, but amyloid PET positivity [HR (95% CI) = 1.58 (0.59–4.24), *p* = 0.363] could not do in the subset with a baseline CDR less than 0.5. We also found that brain age [HR (95% CI) = 2.40 (1.43–4.03), *p* = 0.001] might be a good predictor for the incident dementia unlike MMSE [HR (95% CI) = 1.22 (0.86–1.76), *p* = 0.288], Rt. MTA [HR (95% CI) = 1.57 (0.86–2.88), *p* = 0.143] and Lt. MTA [HR (95% CI) = 1.69 (0.85–3.36), *p* = 0.133] (Supplementary Table 9). In the likelihood ratio tests to compare nested Cox models, the model of brain age residual with amyloid PET positivity showed better fit than those of amyloid PET positivity only (*N* = 366, *χ*^2^ = 12.90, *p* < 0.001; *N* = 284, *χ*^2^ = 12.70, *p* < 0.001; *N* = 240, *χ*^2^ = 13.02, *p* < 0.001). Conversely, the addition of MTA or MMSE could not affect the goodness of fit of brain age residual included Cox model for predicting the incidence dementia [Rt. MTA (*χ*^2^ = 0.18, p = 0.673), Lt. MTA (*χ*^2^ = 0.37, *p* = 0.542), MMSE (*χ*^2^ = 0.00, *p* = 0.992)] (Supplementary Table 10).

**Table 4:** Association of the baseline brain age residual with cognitive decline by Cox proportional hazards models in follow-up participants.

Total participants (*N* = 366)	Cognitive end point: CDR-SB increased at a rate > 2.05 points/year from the baseline^a^ (*n*/*N* = 41/366)			
	HR	95% CI		*p* value (FDR correction)
Model 1	1.71	1.26	2.32	0.001
Model 2	1.95	1.34	2.84	0.001
Model 3	1.97	1.36	2.85	0.001
Model 4	1.94	1.33	2.81	0.001

Participants with baseline CDR ≤ 0.5 (*N* = 284)	Cognitive end point: CDR-SB increased at a rate > 2.05 points/year from the baseline* (*n*/*N* = 22/284)			
	HR	95% CI		*p* value (FDR correction)
Model 1	1.69	1.13	2.54	0.011
Model 2	2.33	1.44	3.76	0.001
Model 3	2.34	1.46	3.75	0.001
Model 4	2.31	1.44	3.71	0.001

Participants with baseline SCD or MCI (*N* = 240)	Cognitive end point: Incident dementia (*n*/*N* = 20/240)			
	HR	95% CI		*p* value (FDR correction)
Model 1	1.93	1.25	2.98	0.003
Model 2	2.43	1.47	4.03	0.001
Model 3	2.49	1.49	4.14	0.001
Model 4	2.40	1.43	4.03	0.001

Brain age residual was calculated as (brain age = intercept + β1[age centred] + β2[age centred squared] + β3[sex] + brain age residual) in these samples.

Model 1: Brain age residual; Model 2: Model 1+ age centred, age centred squared, sex, education, intracranial volume, study site, and baseline CDR-SB; Model 3: Model 2 + APOE e4; Model 4: Model 3 + amyloid PET positive

*HR* hazard ratio *CI*, confidence interval, *CDR-SB* clinical dementia rating sum of box, *PET* positron emission tomography, *APOE* apolipoprotein E

^a^Cognitive end point (time-to-event) was defined when the CDR-SB score increased at a rate >2.05 points/year from the baseline CDR-SB score at the final follow-up [Annual rate of change (slope, 95% CI) in CDR-SB was known as 1.88 (1.77–2.05) in those who progressed to CDR 1 from baseline CDR 0 or 0.5] [23].

## Discussion

We independently validated our previous brain age model results from a clinical perspective, which showed associations between greater brain ageing and amyloid positive status (compared to negative), lower cognitive function, and dementia (compared to MCI or SCD). These data were obtained from a highly different group that included all South Korean individuals, whereas our training data included primarily Caucasian individuals. This replication shows that these effects are generalisable to various samples. We additionally showed that baseline brain age had some benefit in predicting future cognitive decline or incident dementia compared to baseline age, MTA, MMSE, and amyloid levels, indicating that it may be a marker of propensity. Since baseline brain age was predictive of future cognitive decline (even in those without dementia), brain age measurements may be a good tool for monitoring efficacy of preventative approaches and treatment as well as diagnostic prediction. Since our brain age measure is associated with future impairment, this may not only allow for early detection of AD vulnerability, but also allow for much earlier interventions. While the association between age and brain age exhibited a steeper slope for the dementia group (compared to MCI or SCD), this was primarily driven by younger individuals who have dementia. Note, for instance, that younger dementia patients have a much higher brain age and that, in general, dementia patients have a greater brain age.

These results are consistent with those of previous studies applying MR-based brain ageing. Past studies have shown the association between accelerated brain ageing and dementia severity, primarily with prospective decline of cognitive function [29, 30], MCI, and dementia compared to control groups [31], as well as conversion to dementia [7, 32]. Our model previously built on this literature by incorporating amyloid status (i.e. excluding those who had significant amyloid in the brain), we included only individuals who were amyloid negative when training the brain age model [3]. Our previous work [3] showed accelerated ageing in cognitively more impaired or amyloid-positive individuals, which we have now replicated in a completely independent sample. This sample has significantly different characteristics in both ethnicity and the data collection setting than the training set as it includes only South Korean individuals (East Asian); however, the model still reliably predicted brain age and replicated our previous findings that brain age was associated with amyloid status and cognitive function (including cognitive batteries and dementia diagnosis). We now additionally show that in survival analyses the brain age residual was associated with cognitive decline even after adjusting for baseline age, amyloid, and APOE e4 status. Our model outperformed baseline MMSE and MTA as predictors of future decline or incident dementia, showing better performance in a classification in cognitively normal or mildly impairment levels. As expected, brain age was a predictor of current cognition but did not necessarily outperform other metrics but on the other hand, brain age was a better predictor of future cognitive decline. Given that brain age is a holistic measure of neurodegeneration, it is expected that it would most closely tie to risk of cognitive decline since it is temporally further down in the ATN model of AD (thus those with neurodegeneration or low grey matter volumes are at greatest risk).

These results demonstrate that there is potential clinical utility of brain age models in the monitoring of older adults with cognitive impairment, further expanding existing literature [1–3, 30]. Considering that MRI is relatively inexpensive, non-invasive and commonly conducted in patients with cognitive concerns, brain age models may offer benefits in improving the accuracy of clinical diagnosis and informing decision-making in addition to PET imaging and genetic testing. Brain age models, compared to conventional screening tools and visual rating methods, may be informative for predicting future cognitive decline.

Many clinical trials for AD have failed [33], and this may, in part, be attributable to heterogeneous pathology and varying lifestyle and medical factors (e.g. diet, education, mental exertion, leisure participation, multilingualism, sleep, trauma, physical activity, concurrent medications and illnesses) [34]. It has been suggested that future clinical evaluation of AD therapeutics should consider the potential impact of these variables [34], and brain age may act as a holistic measure of multiple processes that converge on neurodegeneration in various ways. ﻿Cole et al. [35] proposed that brain age models automatically place an individual’s brain health in context for their age, summarising complex information regarding neurodegenerative pathology in an intuitive and accessible manner, which could be a key advantage of the brain age paradigm over brain volume or longitudinal atrophy measures. Additionally, Franke and Gaser [1] suggested that this predictive analytical method provides a personalised biomarker of brain structure. This could help to elucidate and further examine the patterns and mechanisms underlying individual differences in brain structure and disease states. Because brain-age estimation is performed on an individual level, the brain age biomarker may be very well suited for clinical use.

### Limitations

There are some limitations that should be considered. Our brain age model incorporates only information from T1-weighted structural scans that focused primarily on grey matter. Diffusion-weighted imaging, fluid-attenuated inversion recovery, and functional imaging are known to change with advancing age and are linked with ageing-related brain disease such as subcortical ischaemia. Integrating these additional data into brain age algorithms may produce biomarkers more predictive of pathogenic brain ageing [1]. We did not evaluate longitudinal brain aging, so it is unclear whether these brain age markers change over time in those with high amyloid (compared to low), AD or MCI (compared to controls), cognitive decline, or those who convert to MCI or AD from controls. In parts of the clinical analysis, the SCD group was used as a de facto control group according to the SCD working group criteria because these participants were from real-world memory clinics [13]. However, SCD might be a contentious category and differ from the cognitively intact state. Thus, the question regarding the utility of this tool in predicting conversion to MCI or dementia in cognitively intact older adults remains. Moreover, we found that the brain age residual of SCD and MCI were negative values, on average, even though the raw gaps between the brain and chronological age had a positive tendency in our samples (Supplementary Table 11). Overall, this validation data showed a higher brain age than chronological age due to the property of cognitively impaired sample. Brain age residual in the regression was a relative location of the fitted value within this dataset with SCD, MCI and dementia. Hence, it should be noted that the negative average of brain age residual in SCD and MCI does not mean ‘younger brain than its chronological age’. Therefore, it is necessary to be careful while interpreting the meaning of the brain age residual (Supplementary Fig. 2). This is a major limitation of the current work as the sample was recruited primarily from clinics/hospital settings though this could also be seen as a strength in a naturalistic setting. In addition, age ranges between training (20–85 years used to train the model) [3] and the test sets (49–89 years) were not exactly matched, which might affect the performance and generalisation of this model. In fact, the SCD and MCI groups showed a negative brain age residual tendency while the dementia group showed a positive value under 80 years of age, but our model was less likely to discriminate the diagnostic group over 80 years of age. Lastly, the data were collected at multiple sites, we accounted for this in our statistical modelling. In some ways, this is a strength of the current result that these results generalise across multiple sites.

## Conclusion

These results from a highly clinical dataset demonstrated that there is potential utility of machine-learning brain age models in the monitoring of cognitive decline and detection of amyloid status in elderly patients. When considering that MRI are commonly conducted in memory clinics, our brain age models may offer benefits for tracking of disease progress, development of preventative approaches and even monitoring treatment.

## Supplementary information

Supplementary material

## Data Availability

The datasets analysed during the current study are available from the corresponding author on reasonable request.
