# Supplementary material for: Independent replication of advanced brain age in mild cognitive impairment and dementia: detection of future cognitive dysfunction
Source: Mol Psychiatry. 2022 Aug 16;27(12):5235–43. doi: 10.1038/s41380-022-01728-y (PMC9763106; doi:10.1038/s41380-022-01728-y)
Supplement: Supplementary file 1 — Supplementary material [file 41380_2022_1728_MOESM1_ESM.docx]

**SUPPLEMENTARY MATERIAL**

**TABLE OF CONTENTS**

**SUPPLEMENTARY MATERIALS AND METHODS** １

**1. Clinical and biological assessment** １

*1.1 Cognitive function* １

*1.2 APOE genotyping* ２

*1.3 Amyloid PET acquisition and measurement of amyloid deposition* ２

*1.4 Neuroimage data and brain age estimation* ２

**SUPPLEMENTARY REFERENCES** ４

**SUPPLEMENTARY TABLES and FIGURE** ７

# **SUPPLEMENTARY MATERIALS AND METHODS**

## **1. Clinical and biological assessment**

### *1.1 Cognitive function*

General cognitive function was evaluated using the Mini Mental Status Examination (MMSE).[1] Dementia severity was measured based on the Clinical Dementia Rating Sum of Box (CDR-SB) score. Cognitive function was evaluated using the Seoul Neuropsychological Screening Battery (SNSB), which includes standardised neuropsychological tests for language, visuospatial abilities, memory, and frontal/executive function as follows:[2] attention ability was evaluated using the Digit Span backward test; language function, based on the Boston Naming Test (BNT); and visuospatial function, based on the Rey Complex Figure Test (RCFT) copy. Memory function was calculated by adding the scores of verbal (Seoul Verbal Learning Test [SVLT]-delayed recall and recognition) and visual (RCFT-delayed recall) memory tests. Frontal/executive function was calculated by adding the scores of the Controlled Oral Word Association Test (COWAT)-animal and Stroop test-color reading. Standard z-scores were obtained for each cognitive test, using a healthy control group as the reference group.[2] Depressive symptoms were evaluated using the Korean version of the short-form Geriatric Depression Scale (SGDS).[3]

### *1.2 APOE genotyping*

Informed consent was obtained from all participants regarding the collection and genotyping of blood genomic DNA. Genomic DNA was isolated from blood samples, and single-nucleotide polymorphism (SNP) genotyping was performed by DNA Link, Inc. (Seoul, Korea) using the Affymetrix Axiom KORV1.0-96 Array (Thermo Fisher Scientific, Waltham, MA, USA) according to the manufacturer’s protocol. APOE genotypes were derived from rs429358 and rs7412, which were included in the array.

### *1.3 Amyloid PET acquisition and measurement of amyloid deposition*

The participants underwent ^18^F-flutemetamol PET scanning using a Discovery STE/690 PET/CT scanner (GE, Milwaukee, WI, USA); the same protocol was used for all participants. ^18^F-flutemetamol was injected into the antecubital vein as a bolus (mean dose, 185 MBq). After 90 min, a 20-min PET scan (4 × 5 min dynamic frames) was performed. ^18^F-flutemetamol PET scans were co-registered to individual MRI scans, which were normalized to a T1-weighted MRI template using transformation parameters. To quantify ^18^F-flutemetamol retention, the standard uptake value ratio (SUVR) was obtained using the pons as a reference region. Global cortical ^18^F-flutemetamol retention was calculated, using the automated anatomical labeling (AAL) atlas, from the volume-weighted average SUVRs of 28 bilateral cortical volumes of interest from the frontal, posterior cingulate, lateral temporal, parietal, and occipital lobes.[4] Based on a previous report on older Koreans and our observed data distribution, participants were considered to be amyloid positive if their global cortical SUVR was greater than 0.65.[5]

### *1.4 Neuroimage data and brain age estimation*

#### 1.4.1 MRI acquisition

MRI scan data were obtained from all participants using a 3.0 T MR scanner. Structural MRI, including 3D T1 and fluid-attenuated inversion recovery (FLAIR), were performed. All MRI images were reviewed by neuroradiologists. The detailed MRI parameters at each site are described in Supplementary Table 1. One site recruited participants in two ways: those who visited the hospital directly and those who were transferred through the community mental health center. Two types of scanners were used according to the recruitment path.

#### 1.4.2 Medial temporal lobe atrophy and WMH rating

To reduce intra- and inter-rater variability, medial temporal lobe atrophy (MTA) and WMH were assessed visually by two psychiatrists and six neurologists who were trained in performing the measurements.[6] The T1 coronal images were used for the visual assessment, and left and right MTA were rated separately. The degree of MTA was rated from 0 (no atrophy) to 4 (severe atrophy).[7] FLAIR images were used to evaluate WMH degrees according to the modified criteria proposed by Fazekas et al.[8] and Scheltens et al.[9] WMH was separately examined in the periventricular and deep white matter lesions. Deep white matter lesion severity was scored as 1 (< 10 mm), 2 (0–25 mm), or 3 (>25 mm) based on the greatest lesion diameter. Periventricular white matter lesion severity was scored as 1 (cap and band <5 mm), 2 (cap and band from 5–10 mm), or 3 (cap and band >10 mm) based on the size of the cap and band, which were perpendicular and horizontal to the ventricle. The degree of overall WMH was classified based on the severity of periventricular and deep WMH as mild (D1P1 and D1P2), moderate (neither mild nor severe: D1P3, D2P1, D2P2, D2P3, D3P1, D3P2), or severe (D3P3).[10]

#### 1.4.3 Structural processing

Processing was conducted using the statistical parametric mapping toolbox (SPM12)[11] in MATLAB (2018b) (MathWorks, Natick, MA, USA). All interpolations were performed with fourth degree B-splines and normalized mutual information similarity metric for registration. T2-SPACE and FLAIR were first independently co-registered with MPRAGE. All three were input into a multispectral segmentation that bias corrects each image and segments them into gray matter, white matter, cerebrospinal fluid, skull, soft tissue, and air.[12] Because of the high burden of WMHs, we adjusted the number of Gaussians used to identify white matter to two to improve the identification of gray and white matter.[13] This ensured an accurate segmentation of the gray matter. The gray and white matter maps were input into a process to generate a study-specific template to estimate the gray matter images. We used Diffeomorphic Anatomical Registration using Exponentiated Lie Algebra (DARTEL) to generate a study-specific template.[14] DARTEL aligned each participant’s gray matter image (along with white matter) to a standard Montreal Neurological Institute (MNI) space template using a combination of linear and nonlinear registrations. DARTEL uses an iterative process of averaging across participants and iterative co-registration to improve normalization to a standard anatomic space. Once a study-specific template was generated (an iterative average across participants), each image preserved the total amount of gray matter by multiplying by the determinant of the Jacobian of the transformations.[14] All images were normalized to a 1-mm^3^ isotropic resolution. The gray matter images were smoothed using a Gaussian kernel of full width at a half-maximum of 4 mm. These gray matter images were input into the brain age estimation model.[15]

# **SUPPLEMENTARY REFERENCES**

1. Oh E, Kang Y, Ph D, Shin JH, Yeon BK. A validity study of K-MMSE as a screening test for dementia : comparison against a comprehensive neuropsychological evaluation. Dement Neurocognitive Disord. 2010;9:8–12.

2. Ahn HJ, Chin J, Park A, Lee BH, Suh MK, Seo SW, et al. Seoul neuropsychological screening battery-dementia version (SNSB-D): a useful tool for assessing and monitoring cognitive impairments in dementia patients. J Korean Med Sci. 2010;25:1071–1076.

3. Bae JN, Cho MJ. Development of the Korean version of the geriatric depression scale and its short form among elderly psychiatric patients. J Psychosom Res. 2004;57:297–305.

4. Tzourio-Mazoyer N, Landeau B, Papathanassiou D, Crivello F, Etard O, Delcroix N, et al. Automated anatomical labeling of activations in SPM using a macroscopic anatomical parcellation of the MNI MRI single-subject brain. Neuroimage. 2002;15:273–289.

5. Hwang J, Jeong JH, Yoon SJ, Park KW, Kim E-JJ, Yoon B, et al. Clinical and biomarker characteristics according to clinical spectrum of Alzheimer’s Disease (AD) in the validation cohort of Korean brain aging study for the early diagnosis and prediction of AD. J Clin Med. 2019;8:341.

6. Yoon B, Shim YS, Cheong HK, Hong YJ, Lee KS, Park KH, et al. White matter hyperintensities in mild cognitive impairment: clinical impact of location and interaction with lacunes and medial temporal atrophy. J Stroke Cerebrovasc Dis. 2014;23:e365–e372.

7. Scheltens P, Leys D, Barkhof F, Huglo D, Weinstein HC, Vermersch P, et al. Atrophy of medial temporal lobes on MRI in ‘probable’ Alzheimer’s disease and normal ageing: diagnostic value and neuropsychological correlates. J Neurol Neurosurg Psychiatry. 1992;55:967–972.

8. Fazekas F, Chawluk JB, Alavi A, Hurtig HI, Zimmerman RA. MR signal abnormalities at 1.5 T in Alzheimer’s dementia and normal aging. Am J Roentgenol. 1987;149:351–356.

9. Scheltens P, Barkhof F, Leys D, Pruvo JP, Nauta JJP, Vermersch P, et al. A semiquantative rating scale for the assessment of signal hyperintensities on magnetic resonance imaging. J Neurol Sci. 1993;114:7–12.

10. Noh Y, Lee Y, Seo SW, Jeong JH, Choi SH, Back JH, et al. A new classification system for ischemia using a combination of deep and periventricular white matter hyperintensities. J Stroke Cerebrovasc Dis. 2014;23:636–642.

11. Penny W, Friston K, Ashburner J, Kiebel S, Nichols T. Statistical Parametric Mapping: The Analysis of Functional Brain Images. Amsterdam: Elsevier; 2011.

12. Ashburner J, Friston KJ. Unified segmentation. Neuroimage. 2005;26:839–851.

13. Karim HT, Andreescu C, MacCloud RL, Butters MA, Reynolds CF, Aizenstein HJ, et al. The effects of white matter disease on the accuracy of automated segmentation. Psychiatry Res - Neuroimaging. 2016;253:7–14.

14. Ashburner J. A fast diffeomorphic image registration algorithm. Neuroimage. 2007;38:95–113.

15. Karim HT, Ly M, Yu G, Krafty R, Tudorascu DL, Aizenstein HJ, et al. Aging faster: worry and rumination in late life are associated with greater brain age. Neurobiol Aging. 2021;101:13–21.

16. Crager MR. Extensions of the absolute standardised hazard ratio and connections with measures of explained variation and variable importance. Lifetime Data Anal. 2020;26:872–892.

17. Williams MM, Storandt M, Roe CM, Morris JC. Progression of Alzheimer’s disease as measured by Clinical Dementia Rating Sum of Boxes scores. Alzheimer’s Dement. 2013;9:39–44.

# **SUPPLEMENTARY TABLES and FIGURE**

**Supplementary Table 1. MRI and PET parameters according to the study site**

|  | **Site** | **Vendor** | **Acquisition matrix** | **voxel size**  **(mm)** | **Repetition time (sec)** | **Echo time (msec)** | **flip angle (⁰)** | **slice thickness (mm)** | **machine** |
| --- | --- | --- | --- | --- | --- | --- | --- | --- | --- |
| **3DT1** | A | [GE] | 256 x 256  512 x 512 | 0.39 x 0.39  0.78 x 0.78 | 7.1-9.9 | 2.8-4.8 | 12 | 1 | GE DISCOVERY MR750w |
|  | B | [Philips] | 480 x 480 | 0.50 x 0.50 | 9.9 | 4.6 | 8 | 1 | Philips Achieva |
|  | C | [GE] | 256 x 256 | 1.00 x 1.00 | 7.4-7.6 | 2.7-2.8 | 11 | 1.2 | GE DISCOVERY MR750w |
|  | D | [GE] | 256 x 256 | 0.88 x 0.88 | 7.5-8.6 | 2.8-3.3 | 12 | 1 | GE DISCOVERY MR750w |
|  | E | [Siemens] | 256 x 256 | 0.98 x 0.98 | 1.8 | 2.1 | 9 | 1 | Siemens TrioTim |
|  | F | [GE] | 512 x 512 | 0.47 x 0.47 | 8.2-14.0 | 3.2-6.0 | 9 | 1.3 | Siemens TrioTim |
|  | G | [Philips] | 480 x 480 | 0.50 x 0.50 | 9.9 | 4.6 | 8 | 1 | Philips Achieva |
| **FLAIR** | A | [GE] | 512 x 512 | 0.39 x 0.39 | 9.7 | 125 | 160 | 5 | GE DISCOVERY MR750w |
|  | B | [Philips] | 512 x 512 | 0.47 x 0.47 | 11 | 125 | 98 | 2 | Philips Achieva |
|  | C | [GE] | 512 x 512 | 0.86 x 0.86 | 9 | 98 | 111 | 6 | GE DISCOVERY MR750w |
|  | D | [GE] | 512 x 512 | 0.39 x 0.39 | 11 | 125 | 173 | 5 | GE DISCOVERY MR750w |
|  | E | [Siemens] | 256 x 224 | 0.82 x 0.82 | 9 | 86 | 130 | 7 | Siemens TrioTim |
|  | F | [GE] | 512 x 512 | 0.45 x 0.45 | 9.5 | 96.8 | 160 | 5.5 | Siemens TrioTim |
|  | G | [Philips] | 512 x 512 | 0.47 x 0.47 | 11 | 125 | 90 | 2 | Philips Achieva |
|  | **Site** | **Vendor** | **Acquisition matrix** | **voxel size (mm)** | **Tracer** | | | | **machine** |
| **PET** | A | [GE] | 128 x 128 x 47 | 1.9531-2, 1.9531-2 | Flutmetamol | | | | GE, Discovery STE |
|  | B | [GE] | 128 x 128 x 188 | 2, 2 | Flutmetamol | | | | GE, Discovery STE |
|  | C | [GE] | 128 x 128 x 47 | 2.344, 2.344 | Flutmetamol | | | | GE, Discovery 690 |
|  | D | [GE] | 128 x 128 x 47 | 1.9531-2, 1.9531-2 | Flutmetamol | | | | GE, Discovery STE/GE, Discovery ST |
|  | E | [Siemens] | 128 x 128 x 148 | 1.9531-2, 1.9531-2 | Flutmetamol | | | | Siemense, Biograph40_TruePoint |
|  | F | [GE] | 256 x 256 x 47 | 0.977, 0.977 | Flutmetamol | | | | GE, Discovery STE |
|  | G | [GE] | 128 x 128 x 188 | 2, 2 | Flutmetamol | | | | GE, Discovery STE |

Abbreviations: MRI, magnetic resonance imaging; PET, positron emission tomography; 3DT1, three-dimensional whole-body T1-weighted; FLAIR, fluid-attenuated inversion recovery

**Supplementary Table 2. Performance metrics calculated in a BICWALZS test set (n=687)^ǂ^**

|  | Total  (n=687) | Diagnosis | | | |
| --- | --- | --- | --- | --- | --- |
|  |  | SCD  (n=80) | MCI  (n=389) | SCD& MCI  (n=469) | Dementia  (n=218) |
| Age year, mean ± SD (range) | 72.55±7.46  (49-89) | 70.16±7.40  (49-84) | 72.50±7.06 (49-89) | 72.10±7.16  (49-89) | 73.51±8.00 (51-88) |
| MAE, mean ± SD | 5.63± S4.71 | 4.89±3.53 | 5.05±4.08 | 5.02±3.99 | 6.95±5.76 |
| RMSE, mean ± SD | 7.35±9.57 | 6.03±6.71 | 6.49±8.20 | 6.41±8.00 | 9.03±11.34 |
| r | 0.47* | 0.64* | 0.55* | 0.57* | 0.24* |
| R2 | 0.22 | 0.41 | 0.30 | 0.33 | 0.06 |

ǂ Brain age was calculated using the data of 687 participant with 3DT1 weighted MRI from BICWALZS

* *p*<0.001

Abbreviations: MRI, magnetic resonance imaging; BICWALZS, Biobank Innovations for chronic Cerebrovascular disease With ALZheimer's disease Study; SD, standard deviation; SCD, subjective cognitive decline; MCI, mild cognitive impairment; MAE, mean absolute error; RMSE, root mean square error

**Supplementary Table 3. Characteristics of study participants in neurocognitive tests and MRI visual rating scales**

|  | Total baseline sample (N=650) | | Number missing |
| --- | --- | --- | --- |
|  | Mean or n | SD or % |  |
| MMSE score, mean (SD) | 23.11 | 5.22 | 8 |
| SGDS score, mean (SD) | 6.24 | 5.09 | 3 |
| Neurocognitive Test Z score, mean (SD) |  |  |  |
| Digit span-backward | -0.57 | 1.16 | 12 |
| Boston Naming Test | -0.82 | 1.65 | 12 |
| RCFT-copy | -1.23 | 2.23 | 9 |
| RCFT-delayed recall | -0.82 | 1.12 | 18 |
| RCFT-recognition | -0.93 | 1.35 | 26 |
| SVLT-delayed recall | -1.15 | 1.22 | 10 |
| SVLT-recognition | -1.11 | 1.53 | 10 |
| COWAT | -0.90 | 1.16 | 28 |
| Stroop test-color reading | -1.08 | 1.55 | 42 |
| Study site, n (%) |  |  | 0 |
| Hospital 1 | 239 | 36.80 |  |
| Hospital 2 | 91 | 14.00 |  |
| Hospital 3 | 93 | 14.30 |  |
| Hospital 4 | 28 | 4.30 |  |
| Hospital 5 | 18 | 2.80 |  |
| Hospital 6 | 165 | 25.40 |  |
| Hospital 7 | 16 | 2.50 |  |
| Lacunae, n (%) | 1.61 | 3.11 | 0 |
| WMH severity, n (%) |  |  | 0 |
| Mild | 386 | 59.40 |  |
| Moderate | 214 | 32.90 |  |
| Severe | 50 | 7.70 |  |
| MTA score_Rt, n (%) |  |  | 0 |
| 0 | 53 | 8.20 |  |
| 1 | 216 | 33.20 |  |
| 2 | 259 | 39.80 |  |
| 3 | 103 | 15.80 |  |
| 4 | 19 | 2.90 |  |
| MTA score_Lt, n (%) |  |  | 0 |
| 0 | 40 | 6.20 |  |
| 1 | 196 | 30.20 |  |
| 2 | 275 | 42.30 |  |
| 3 | 116 | 17.80 |  |
| 4 | 23 | 3.50 |  |
| Intracranial volume, mean (SD) | 1675.14 | 928.90 | 0 |

Abbreviations: MMSE, Mini Mental Status Examination; SGDS, short-form Geriatric Depression Scale; RCFT, Rey Complex Figure Test; SVLT, Seoul Verbal Learning Test; COWAT, Controlled Oral Word Association Test; WMH, white matter hyperintensities; MTA, medial temporal lobe atrophy

**Supplementary Table 4. Linear regression analysis for associations of brain age residual and cognitive function**

| Dependent variables | Independent variable: brain age residual | | | | | | |
| --- | --- | --- | --- | --- | --- | --- | --- |
|  | **n** | **β** | **B** | **SE** | **95% CI** | | **p value** |
| General cognition (score) |  |  |  |  |  | |  |
| CDR-Sum of Box | 650 | 0.28 | 0.77 | 0.10 | 0.58 | 0.96 | <0.001 |
| MMSE | 642 | -0.23 | -1.18 | 0.18 | -1.53 | -0.83 | <0.001 |
| Neurocognitive test (z-score) |  |  |  |  |  |  |  |
| Digit span-backward | 638 | -0.15 | -0.13 | 0.05 | -0.24 | -0.06 | 0.001 |
| Boston naming test | 638 | -0.21 | -0.35 | 0.06 | -0.48 | -0.23 | <0.001 |
| RCFT-copy | 641 | -0.26 | -0.57 | 0.08 | -0.73 | -0.41 | <0.001 |
| RCFT-delayed recall | 632 | -0.23 | -0.20 | 0.04 | -0.31 | -0.15 | <0.001 |
| RCFT-recognition | 624 | -0.20 | -0.14 | 0.05 | -0.30 | -0.09 | <0.001 |
| SVLT-delayed recall | 640 | -0.18 | -0.15 | 0.04 | -0.26 | -0.10 | <0.001 |
| SVLT-recognition | 640 | -0.29 | -0.19 | 0.06 | -0.40 | -0.18 | <0.001 |
| COWAT | 622 | -0.26 | -0.22 | 0.05 | -0.35 | -0.17 | <0.001 |
| Stroop test-color reading | 608 | -0.26 | -0.41 | 0.06 | -0.52 | -0.30 | <0.001 |

Brain age residual was calculated as (brain age = intercept + β1[age centred] + β2[age centred squared] + β3[sex] + brain age residual), and a linear regression model was developed after adjustment for age centred, age centred squared, sex, education, intracranial volume, study site, amyloid PET positivity, and APOE e4.A separate regression was conducted for each cognitive test.

Abbreviations: SE, standard error; CI, confidence interval; MMSE, Mini Mental Status Examination; RCFT, Rey Complex Figure Test; SVLT, Seoul Verbal Learning Test; COWAT, Controlled Oral Word Association Test; APOE, apolipoprotein E

**Supplementary Table 5. Regression models explaining variance in brain age**

| Variables (N=650) | β | B | SE | 95% CI | P value |
| --- | --- | --- | --- | --- | --- |
| Age | 1.05 | 0.72 | 0.34 | (0.06, 1.38) | 0.034 |
| Age^2^ | -0.59 | 0.00 | 0.00 | (-0.01, 0.00) | 0.235 |
| Education | -0.05 | -0.05 | 0.04 | (-0.13, 0.03) | 0.216 |
| Sex (Female reference) | 0.25 | 2.71 | 0.39 | (1.94, 3.48) | <0.001 |
| Study site | -0.07 | -0.18 | 0.09 | (-0.13, 0.03) | 0.216 |
| APOE e4  (e4 negative reference) | -0.05 | -0.60 | 0.40 | (-1.38, 0.19) | 0.136 |
| Amyloid PET positive  (Negative reference) | 0.09 | 0.91 | 0.38 | (0.14, 1.69) | 0.021 |
| WMH severity (visual rating) | 0.00 | 0.00 | 0.29 | (-0.57, 0.56) | 0.991 |
| Lacunae number | 0.02 | 0.02 | 0.06 | (-0.09, 0.14) | 0.674 |

Linear regression model. β values indicate standardised coefficients while B indicates unstandardised coefficients.

Abbreviations: APOE, apolipoprotein E; WMH, white matter hyperintensities

**Supplementary Table 6. AUC, sensitivity, and specificity of the brain age residual, amyloid PET SUVR, and MMSE for diagnosis of dementia according to participant age**

|  | AUC (SE, 95% CI) | Cut-off | Sensitivity | Specificity |
| --- | --- | --- | --- | --- |
| Total, Age 49-89 year  (Dementia/Non-dementia: N=204/446) |  |  |  |  |
| MMSE only (Model 1) | 0.876 (0.015,0.846-0.906) | 0.691 | 0.779 | 0.838 |
| Amyloid PET SUVR only (Model 2) | 0.785 (0.019,0.747-0.823) | 0.626 | 0.805 | 0.672 |
| Brain age residual only (Model 3) | 0.761 (0.020,0.722-0.800) | 0.665 | 0.686 | 0.716 |
| Amyloid PET SUVR + MMSE (Model 4) | 0.884 (0.015,0.855-0.913) | 0.699 | 0.761 | 0.874 |
| Brain age residual + MMSE (Model 5) | 0.879 (0.015,0.850-0.909) | 0.661 | 0.811 | 0.818 |
| Brain age residual +Amyloid PET SUVR + MMSE (Model 6) | 0.886 (0.015,0.858-0.915) | 0.647 | 0.815 | 0.828 |
| Age ≤ 77 years  (Dementia/Non-dementia: N=) |  |  |  |  |
| MMSE only (Model 1) | 0.872 (0.021,0.832-0.912) | 0.717 | 0.793 | 0.832 |
| Amyloid PET SUVR only (Model 2) | 0.799 (0.024,0.753-0.846) | 0.741 | 0.735 | 0.754 |
| Brain age residual only (Model 3) | 0.782 (0.025,0.733-0.830) | 0.579 | 0.871 | 0.566 |
| Amyloid PET SUVR + MMSE (Model 4) | 0.884 (0.019,0.847-0.921) | 0.716 | 0.816 | 0.857 |
| Brain age residual + MMSE (Model 5) | 0.878 (0.020,0.838-0.917) | 0.685 | 0.845 | 0.798 |
| Brain age residual +Amyloid PET SUVR + MMSE (Model 6) | 0.887 (0.019,0.849-0.924) | 0.714 | 0.812 | 0.866 |
| Age ≤ 70 years  (Dementia/Non-dementia: N=67/167) |  |  |  |  |
| MMSE only (Model 1) | 0.912 (0.027,0.860-0.965) | 0.697 | 0.878 | 0.885 |
| Amyloid PET SUVR only (Model 2) | 0.872 (0.028,0.817-0.928) | 0.707 | 0.797 | 0.823 |
| Brain age residual only (Model 3) | 0.870 (0.028,0.814-0.926) | 0.604 | 0.845 | 0.774 |
| Amyloid PET SUVR + MMSE (Model 4) | 0.928 (0.024,0.881-0.975) | 0.708 | 0.865 | 0.934 |
| Brain age residual + MMSE (Model 5) | 0.921 (0.024,0.874-0.968) | 0.658 | 0.899 | 0.852 |
| Brain age residual +Amyloid PET SUVR + MMSE (Model 6) | 0.937 (0.022,0.895-0.980) | 0.671 | 0.905 | 0.918 |

Brain age residual was calculated as (brain age = intercept + β1[age centred] + β2[age centred squared] + β3[sex] + brain age residual).

Model 1: MMSE + age centred, age centred squared, sex, education, intracranial volume, study site and APOE e4; Model 2: Amyloid PET SUVR + age centred, age centred squared, sex, education, intracranial volume, study site and APOE e4; Model 3: Brain age residual + age centred, age centred squared, sex, education, intracranial volume, study site and APOE e4; Model 4: Model 1 + Amyloid PET SUVR; Model 5: Model 1 + Brain age residual; Model 6: Amyloid PET SUVR + Brain age residual

Abbreviations: AUC, area under the curve; PET, positron emission tomography; SUVR, standardised uptake value ratio; MMSE, Mini Mental Status Examination; SE, standard error; CI, confidence interval

**Supplementary Table 7. Baseline clinical characteristics of study participants with follow-up**

|  | Follow-up participants  (Baseline SCD or MCI, n=240) | | Follow-up participants  (Baseline CDR ≤0.5, n=284) | | Follow-up participants  (Total, n=366) | |
| --- | --- | --- | --- | --- | --- | --- |
|  | Mean or n | SD or % | Mean or n | SD or % | Mean or n | SD or % |
| Brain age, mean (SD), years | 74.71 | 5.23 | 74.81 | 5.18 | 75.49 | 5.18 |
| Age, mean (SD), years | 72.61 | 7.01 | 72.82 | 7.23 | 72.63 | 7.46 |
| Education, mean (SD), years | 8.24 | 4.99 | 8.24 | 4.97 | 8.15 | 5.11 |
| Female, n (%) | 159 | 66.30 | 187 | 65.80 | 244 | 66.70 |
| Comorbidity, n (%) |  |  |  |  |  |  |
| Hypertension | 115 | 47.90 | 139 | 48.90 | 191 | 52.20 |
| Diabetes mellitus | 49 | 20.40 | 58 | 20.40 | 76 | 20.80 |
| Hyperlipidemia | 98 | 40.80 | 113 | 39.80 | 142 | 38.80 |
| Cardiovascular Disease | 18 | 7.50 | 20 | 7.00 | 24 | 6.60 |
| CDR, n (%) |  |  |  |  |  |  |
| 0 | 5 | 2.10 | 5 | 1.80 | 5 | 1.40 |
| 0.5 | 235 | 97.90 | 279 | 98.20 | 279 | 76.20 |
| 1 | 0 | 0 | 0 | 0 | 69 | 18.90 |
| 2 or more | 0 | 0 | 0 | 0 | 13 | 3.50 |
| CDR-Sum of Box score, mean (SD) | 1.64 | 0.94 | 1.83 | 1.04 | 2.91 | 2.60 |
| Clinical diagnosis, n (%) |  |  |  |  |  |  |
| SCD | 32 | 13.30 | 32 | 11.30 | 32 | 8.74 |
| MCI | 208 | 86.70 | 208 | 73.20 | 219 | 59.84 |
| AD | 0 | 0 | 29 | 10.20 | 73 | 19.95 |
| SVaD | 0 | 0 | 9 | 3.20 | 28 | 7.65 |
| Other dementia | 0 | 0 | 6 | 2.20 | 14 | 3.82 |
| APOE genotype, n (%) |  |  |  |  |  |  |
| E2/E2 | 0 | 0 | 0 | 0 | 0 | 0 |
| E3/E2 | 35 | 14.60 | 37 | 13.00 | 47 | 12.80 |
| E3/E3 | 151 | 62.90 | 177 | 62.30 | 210 | 57.40 |
| E4/E2 | 6 | 2.50 | 7 | 2.50 | 10 | 2.70 |
| E4/E3 | 47 | 19.60 | 59 | 20.80 | 87 | 23.80 |
| E4/E4 | 1 | 0.40 | 4 | 1.40 | 12 | 3.30 |
| Amyloid PET positive, n (%) | 71 | 29.60 | 96 | 33.80 | 148 | 40.40 |
| Global amyloid SUVR score, mean (SD) | 0.65 | 0.14 | 0.67 | 0.15 | 0.70 | 0.17 |
| Follow-up duration, mean (SD), months | 19.44 | 8.47 | 19.38 | 8.44 | 19.69 | 8.66 |

Abbreviations: SD, standard deviation; CDR, clinical dementia rating; SCD, subjective cognitive decline; MCI, mild cognitive impairment; AD, Alzheimer’s disease; SVaD, subcortical vascular dementia; APOE, apolipoprotein E; PET, positron emission tomography; SUVR, standardised uptake value ratio

**Supplementary Table 8. Baseline characteristics of study participants with follow-up data for neurocognitive tests and MRI visual rating scales**

|  | Subset of follow-up participants  (Baseline SCD or MCI, n=240) | | | Subset of follow-up participants  (Baseline CDR ≤0.5, n=284) | | | Follow-up participants  (Total, n=366) | | | |
| --- | --- | --- | --- | --- | --- | --- | --- | --- | --- | --- |
|  | Mean or N | SD or % | Missing N | Mean or N | SD or % | Missing N | Mean or N | SD or % | Missing N |  |
| MMSE score, mean (SD) | 25.32 | 3.50 | 2 | 24.82 | 3.77 | 2 | 23.36 | 5.16 | 6 |  |
| SGDS score, mean (SD) | 6.48 | 5.01 | 1 | 6.38 | 4.97 | 1 | 6.11 | 4.93 | 2 |  |
| Neurocognitive test Z score, mean (SD) |  |  |  |  |  |  |  |  |  |  |
| Digit span-backward | -0.37 | 0.95 | 2 | -0.41 | 0.94 | 2 | -0.57 | 1.05 | 8 |  |
| Boston Naming Test | -0.35 | 1.18 | 2 | -0.46 | 1.30 | 2 | -0.80 | 1.53 | 8 |  |
| RCFT-copy | -0.67 | 1.46 | 2 | -0.77 | 1.61 | 2 | -1.20 | 1.99 | 6 |  |
| RCFT-delayed recall | -0.51 | 1.04 | 3 | -0.61 | 1.07 | 4 | -0.83 | 1.09 | 11 |  |
| RCFT-recognition | -0.45 | 0.98 | 10 | -0.55 | 1.03 | 11 | -0.89 | 1.27 | 17 |  |
| SVLT-delayed recall | -0.78 | 1.19 | 2 | -0.91 | 1.21 | 2 | -1.15 | 1.21 | 7 |  |
| SVLT-recognition | -0.68 | 1.33 | 2 | -0.81 | 1.39 | 2 | -1.16 | 1.56 | 7 |  |
| COWAT | -0.49 | 1.00 | 9 | -0.58 | 1.05 | 14 | -0.75 | 1.03 | 25 |  |
| Stroop test-color reading | -0.62 | 1.16 | 8 | -0.74 | 1.22 | 10 | -1.07 | 1.43 | 27 |  |
| Study site, n (%) |  |  | 0 |  |  | 0 |  |  | 0 |  |
| Hospital 1 | 100 | 41.70 |  | 111 | 39.10 |  | 165 | 45.10 |  |  |
| Hospital 2 | 37 | 15.40 |  | 47 | 16.50 |  | 59 | 16.10 |  |  |
| Hospital 3 | 28 | 11.70 |  | 35 | 12.30 |  | 37 | 10.10 |  |  |
| Hospital 4 | 6 | 2.50 |  | 8 | 2.80 |  | 12 | 3.30 |  |  |
| Hospital 5 | 0 | 0 |  | 4 | 1.40 |  | 7 | 1.90 |  |  |
| Hospital 6 | 69 | 28.80 |  | 79 | 27.80 |  | 86 | 12.50 |  |  |
| Hospital 7 | 0 | 0 |  | 0 | 0 |  | 0 | 0 |  |  |
| Lacunae number, mean (SD) | 1.02 | 2.24 | 0 | 1.10 | 2.33 | 0 | 1.20 | 2.33 | 0 |  |
| WMH severity, n (%) |  |  | 0 |  |  | 0 |  |  | 0 |  |
| Mild | 154 | 64.20 |  | 172 | 60.60 |  | 217 | 59.30 |  |  |
| Moderate | 73 | 30.40 |  | 92 | 32.40 |  | 117 | 32.00 |  |  |
| Severe | 13 | 5.40 |  | 20 | 7.00 |  | 32 | 8.70 |  |  |
| MTA score_Rt, n (%) |  |  | 0 |  |  | 0 |  |  | 0 |  |
| 0 | 27 | 11.30 |  | 28 | 9.90 |  | 28 | 7.70 |  |  |
| 1 | 99 | 41.30 |  | 106 | 37.30 |  | 122 | 33.30 |  |  |
| 2 | 91 | 37.90 |  | 113 | 39.80 |  | 145 | 39.60 |  |  |
| 3 | 23 | 9.60 |  | 35 | 12.30 |  | 62 | 16.90 |  |  |
| 4 | 0 | 0 |  | 2 | 0.70 |  | 9 | 2.50 |  |  |
| MTA score_Lt, n (%) |  |  | 0 |  |  | 0 |  |  | 0 |  |
| 0 | 19 | 7.90 |  | 20 | 7.00 |  | 20 | 5.50 |  |  |
| 1 | 90 | 37.50 |  | 96 | 33.80 |  | 111 | 30.30 |  |  |
| 2 | 107 | 44.60 |  | 128 | 45.10 |  | 162 | 44.30 |  |  |
| 3 | 24 | 10.00 |  | 38 | 13.40 |  | 65 | 17.80 |  |  |
| 4 | 0 | 0 |  | 2 | 0.70 |  | 8 | 2.20 |  |  |
| Intracranial volume, mean (SD) | 1775.88 | 548.74 | 0 | 1805.50 | 575.39 | 0 | 1791.47 | 579.37 | 0 |  |

Abbreviations: MMSE, Mini Mental Status Examination; SGDS, short-form Geriatric Depression Scale; RCFT, Rey Complex Figure Test; SVLT, Seoul Verbal Learning Test; COWAT, Controlled Oral Word Association Test; WMH, white matter hyperintensities; MTA, medial temporal lobe atrophy

**Supplementary Table 9. Comparison of the absolute standardised hazard ratio for cognitive decline among baseline brain age residual, MTA score, and MMSE in the total follow-up participants^†^**

| Total participants (N=366) | Cognitive end point:  CDR-SB increased at a rate >2.05 points/year from the baseline^*^  (n/N= 41/366) | | | |
| --- | --- | --- | --- | --- |
|  | **HR** | **95% CI** | | **p value** |
| Brain age residual (continuous)^ǂ^ | 1.94 | 1.33 | 2.81 | 0.001 |
| Brain age residual (dichotomized: >median vs. <median)^ǂ^ | 2.24 | 1.07 | 4.70 | 0.033 |
| Baseline hippocampus volume (MTA-visual rating scale_Rt)^ǂ^ | 1.92 | 1.29 | 2.84 | 0.001 |
| Baseline hippocampus volume (MTA-visual rating scale_Lt)^ǂ^ | 1.89 | 1.25 | 2.88 | 0.003 |
| Baseline MMSE^ǂ^ | 0.96 | 0.68 | 1.35 | 0.801 |
| Amyloid PET positivity (dichotomized: >SUVR 0.65 vs. < SUVR 0.65)^ǂǂ^ | 2.15 | 0.98 | 4.73 | 0.056 |
| Participants with baseline CDR ≤0.5 (N=284) | **Cognitive end point:**  **CDR-SB increased at a rate >2.05 points/year from the baseline***  **(n/N= 22/284)** | | | |
|  | **HR** | **95% CI** | | **p value** |
| Brain age residual (continuous)^ǂ^ | 2.31 | 1.44 | 3.71 | 0.001 |
| Brain age residual (dichotomized: >median vs. <median)^ǂ^ | 3.68 | 1.35 | 10.01 | 0.011 |
| Baseline hippocampus volume (MTA-visual rating scale_Rt)^ǂ^ | 2.15 | 1.20 | 3.83 | 0.010 |
| Baseline hippocampus volume (MTA-visual rating scale_Lt)^ǂ^ | 1.75 | 0.99 | 3.11 | 0.055 |
| Baseline MMSE^ǂ^ | 1.00 | 0.72 | 1.39 | 0.997 |
| Amyloid PET positivity (dichotomized: >SUVR 0.65 vs. < SUVR 0.65)^ǂǂ^ | 1.58 | 0.59 | 4.24 | 0.363 |
| Participants with baseline SCD or MCI (N=240) | **Cognitive end point:**  **Incident dementia**  **(n/N= 20/240)** | | | |
|  | **HR** | **95% CI** | | **p value** |
| Brain age residual (continuous)^ǂ^ | 2.40 | 1.43 | 4.03 | 0.001 |
| Brain age residual (dichotomized: >median vs. <median)^ǂ^ | 15.90 | 4.09 | 61.88 | <.0001 |
| Baseline hippocampus volume (MTA-visual rating scale_Rt)^ǂ^ | 1.57 | 0.86 | 2.88 | 0.143 |
| Baseline hippocampus volume (MTA-visual rating scale_Lt)^ǂ^ | 1.69 | 0.85 | 3.36 | 0.133 |
| Baseline MMSE^ǂ^ | 1.22 | 0.85 | 1.76 | 0.288 |
| Amyloid PET positivity (dichotomized: >SUVR 0.65 vs. < SUVR 0.65)^ǂǂ^ | 2.74 | 0.86 | 8.70 | 0.088 |

Brain age residual was calculated as (brain age = intercept + β1[age centred] + β2[age centred squared] + β3[sex] + brain age residual) in these samples.

† Absolute standardised hazard ratios were calculated for making comparison among different measures.[16]

ǂ Cox proportional hazards models were conducted in the follow-up participants adjusting for age centred, age centred squared, sex, education, intracranial volume, study site, baseline CDR-SB, APOE e4, and amyloid PET positivity.

ǂǂ Cox proportional hazards models were conducted in the follow-up participants adjusting for age centred, age centred squared, sex, education, study site, baseline CDR-SB, and APOE e4.

*Cognitive end point (time-to-event) was defined when the CDR-SB score increased at a rate >2.05 points/year from the baseline CDR-SB score at the final follow-up (Annual rate of change (slope, 95% CI) in CDR-SB was known as 1.88(1.77-2.05) in those who progressed to CDR 1 from baseline CDR 0 or 0.5).[17]

Abbreviations: HR, hazard ratio; CI, confidence interval; CDR-SB, Clinical Dementia Rating Sum of Box; APOE, apolipoprotein E; MTA, medial temporal lobe atrophy; MMSE, Mini Mental Status Examination; PET, positron emission tomography

**Supplementary Table 10. Likelihood ratio tests to compare Cox models**

| Total participants (N=366) | Log likelihood | df | χ^2^ | p value |
| --- | --- | --- | --- | --- |
| Model A [Baseline Amyloid PET positivity^ǂ^] vs. | -197.84 |  |  |  |
| Baseline Brain age residual (continuous )+ Model A | -191.39 | 1 | 12.90 | <0.001 |
| Model B [Baseline Brain age residual (continuous )+ Model A] vs. | -191.39 |  |  |  |
| Baseline MTA-visual rating scale_Rt + Model B | -188.71 | 1 | 5.36 | 0.021 |
| Baseline MTA-visual rating scale_Lt + Model B | -188.91 | 1 | 4.97 | 0.026 |
| Baseline MMSE+ Model B | -191.27 | 1 | 0.24 | 0.623 |
| Participants with baseline CDR ≤0.5 (N=284) | **Log likelihood** | **df** | **χ^2^** | **p value** |
| Model A [Baseline Amyloid PET positivity^ǂ^] vs. | -95.73 |  |  |  |
| Baseline Brain age residual (continuous )+ Model A | -89.38 | 1 | 12.70 | <0.001 |
| Model B [Baseline Brain age residual (continuous )+ Model A] vs. | -89.38 |  |  |  |
| Baseline MTA-visual rating scale_Rt + Model B | -87.89 | 1 | 2.98 | 0.085 |
| Baseline MTA-visual rating scale_Lt + Model B | -88.76 | 1 | 1.23 | 0.267 |
| Baseline MMSE+ Model B | -88.91 | 1 | 0.95 | 0.330 |
| Participants with baseline SCD or MCI (N=240) | **Log likelihood** | **df** | **χ^2^** | **p value** |
| Model A [Baseline Amyloid PET positivity^ǂ^] vs. | -70.74 |  |  |  |
| Baseline Brain age residual (continuous )+ Model A | -64.23 | 1 | 13.02 | <0.001 |
| Model B [Baseline Brain age residual (continuous )+ Model A] vs. | -64.23 |  |  |  |
| Baseline MTA-visual rating scale_Rt + Model B | -64.14 | 1 | 0.18 | 0.673 |
| Baseline MTA-visual rating scale_Lt + Model B | -64.04 | 1 | 0.37 | 0.542 |
| Baseline MMSE+ Model B | -64.23 | 1 | 0.00 | 0.992 |

Brain age residual was calculated as (brain age = intercept + β1[age centred] + β2[age centred squared] + β3[sex] + brain age residual) in these samples.

ǂ Cox proportional hazards models were conducted in the follow-up participants adjusting for age centred, age centred squared, sex, education, intracranial volume, study site, baseline CDR-SB, and APOE e4.

Abbreviations: MMSE, Mini Mental Status Examination; PET, positron emission tomography; CDR-SB, Clinical Dementia Rating Sum of Box; APOE, apolipoprotein E; MTA, medial temporal lobe atrophy

**Supplementary Table 11. Characteristics of brain age residual and difference between brain and chronological age**

|  | **Diagnosis** | **N** | **Mean** | **Standard Deviation** | **95% Confidence Interval** | |
| --- | --- | --- | --- | --- | --- | --- |
| **Brain Age residual** | SCD | 71 | -0.50 | 0.89 | -0.71 | -0.29 |
|  | MCI | 375 | -0.08 | 0.92 | -0.18 | 0.01 |
|  | Dementia | 204 | 0.33 | 1.08 | 0.18 | 0.48 |
|  | Total | 650 | 0.00 | 1.00 | -0.77 | 0.77 |
| **Difference between Brain and Chronological Age** | SCD | 71 | 2.28 | 5.86 | 0.90 | 3.67 |
|  | MCI | 375 | 2.47 | 6.04 | 1.86 | 3.09 |
|  | Dementia | 204 | 3.73 | 8.31 | 2.58 | 4.88 |
|  | Total | 650 | 2.85 | 6.83 | 2.32 | 3.37 |

Brain age residual was calculated as (brain age = intercept + β1[age centred] + β2[age centred squared] + β3[sex] + brain age residual).

Abbreviations: SCD, subjective cognitive decline; MCI, mild cognitive impairment

**Supplementary Figure 1. The correlation of brain age residual with grey matter volume using voxel-wise analysis**

**
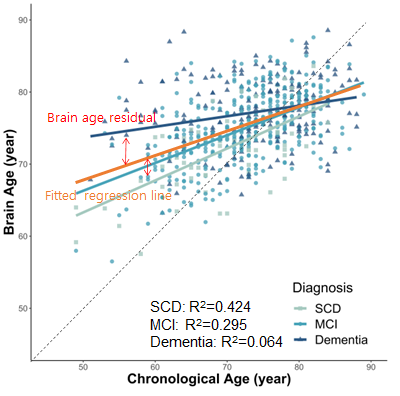
**

Brain age residual was calculated as (brain age = intercept + β1[age centred] + β2[age centred squared] + β3[sex] + brain age residual). Voxel-wise analysis in Statistical nonParametric Mapping between brain age residual and grey matter density was conducted. The red parts mean ‘greater brain age residual is associated with greater grey matter volume (GMV).’ Whereas the blue parts mean ‘greater brain age residual is associated with lower GMV. Multiple comparisons were adjusted by family-wise error correction.

**Supplementary Figure 2. Association between brain age and chronological age according to clinical diagnosis at baseline in all participants**

**
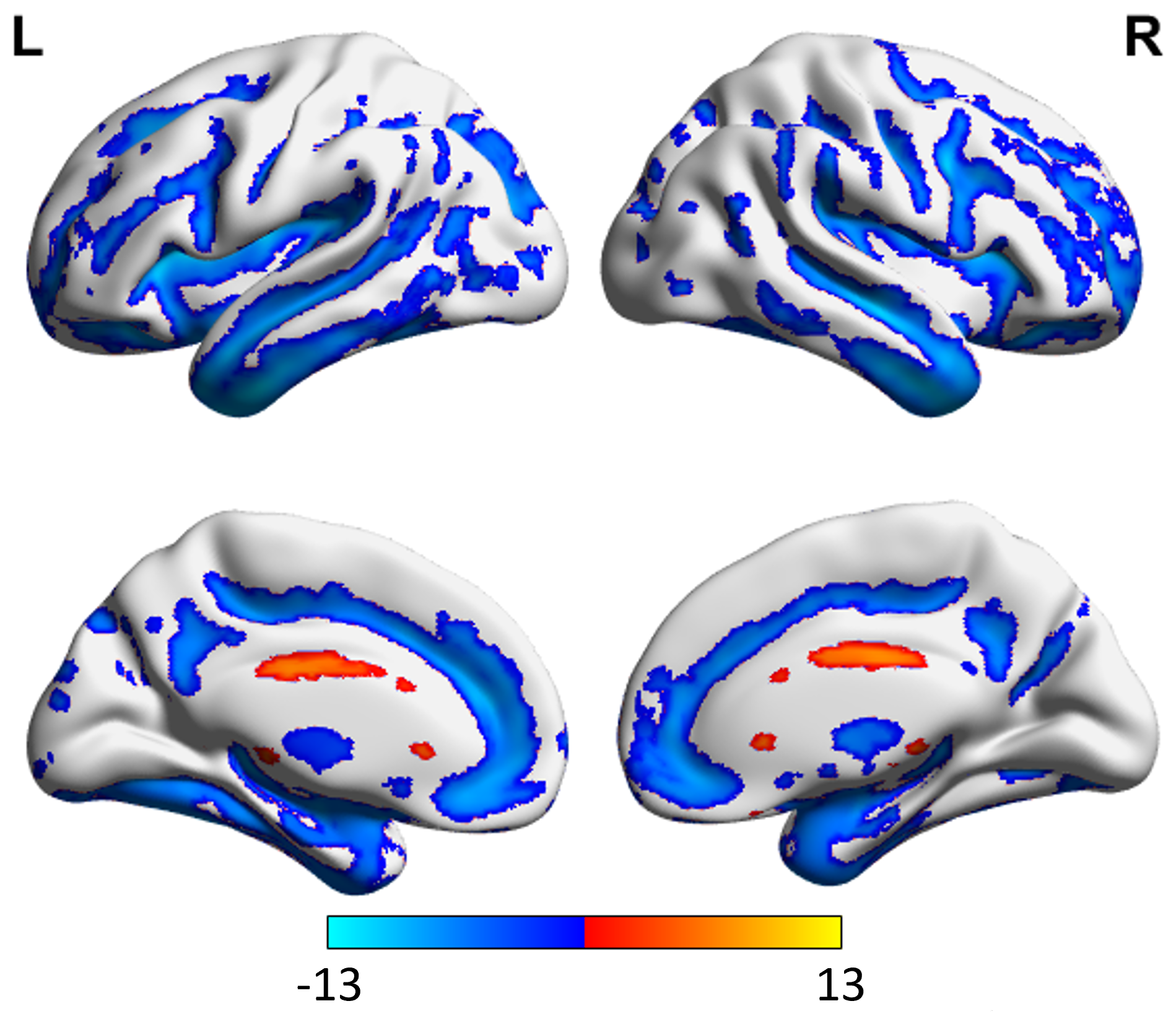
**

Abbreviations: SCD, subjective cognitive decline; MCI, mild cognitive impairment

**Supplementary Figure 3. Association between brain age and chronological age according to sex and white matter hyperintensities at baseline in all participants**

**
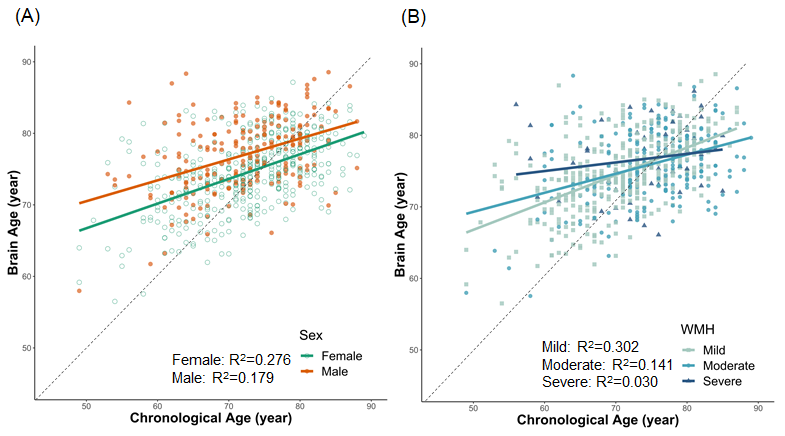
**

(A) Association between brain and chronological ages according to sex. (B) Association between brain and chronological ages according to the severity of WMH.

Abbreviations: WMH, white matter hyperintensities

**Supplementary Figure 4. ROC curve analysis of the brain age residual, amyloid PET SUVR, and MMSE for the diagnosis of dementia according to participant age**


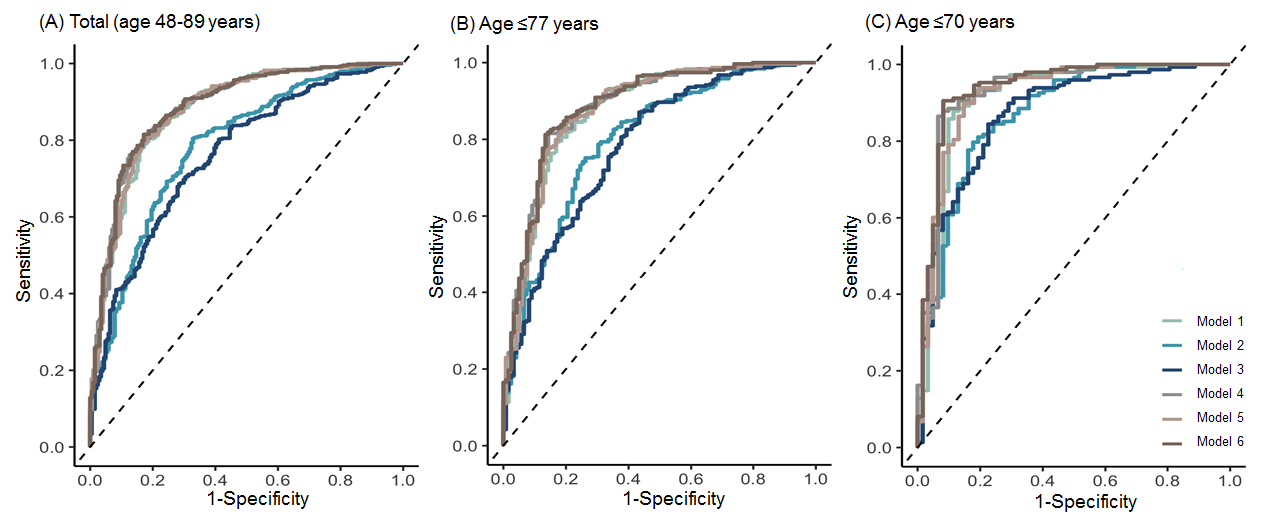


Brain age residual was calculated as (brain age = intercept + β1[age centred] + β2[age centred squared] + β3[sex] + brain age residual).

Model 1: MMSE + age centred, age centred squared, sex, education, intracranial volume, study site and APOE e4; Model 2: Amyloid PET SUVR + age centred, age centred squared, sex, education, intracranial volume, study site and APOE e4; Model 3: Brain age residual + age centred, age centred squared, sex, education, intracranial volume, study site and APOE e4; Model 4: Model 1 + Amyloid PET SUVR; Model 5: Model 1 + Brain age residual; Model 6: Amyloid PET SUVR + Brain age residual
